# The Toxicity of *Eichhornia crassipes* Fractionated Extracts against *Aphis craccivora* and Its Safety in Albino Rats

**DOI:** 10.3390/toxins14050327

**Published:** 2022-05-04

**Authors:** Sara Taha Abdelkhalek, Sherein Saied Abdelgayed, Hong Jiang, Man-Qun Wang

**Affiliations:** 1 Hubei Insect Resources Utilization and Sustainable Pest Management Key Laboratory, College of Plant Science and Technology, Huazhong Agricultural University, Wuhan 430070, China; staha2015@yahoo.com; 2 Department of Entomology, Faculty of Science, Ain Shams University, Cairo 11566, Egypt; 3 Department of Pathology, Faculty of Veterinary Medicine, Cairo University, Giza 12211, Egypt; sherein.abdelgayed@vet.cu.edu.eg; 4 Department of Chemistry, College of Science, Huazhong Agricultural University, Wuhan 430070, China; jianghong0066@126.com

**Keywords:** acetone extract of *E. crassipes*, ethanol extract of *E. crassipes*, GC/MS analysis, bean aphid, bioassay, hematological indices, biochemical assessment, histopathology

## Abstract

*Eichhornia crassipes* were evaluated in order to investigate the insecticidal activity towards *Aphis craccivora* adults. The LC_50_ values were promising and reflected the bio-efficacy of the tested extracts (39 and 42 mg/L), respectively, and reduced the fecundity markedly. Using GC/MS analysis, the major components were n-hexadecanoic, linolenic, hexadecenoic, myristic, stearic acids, linolelaidic acid, methyl ester and some terpenoids, alkaloids, and hydrocarbons. A safety assessment of non-target organisms is essential for the development of new pesticides. In order to guide the rational use of the most potential insecticidal extracts AcF and EtF, the effect of these extracts on body weight, hematological indices, biochemical indicators, and histopathology of some relevant organs of albino rats (as a model for mammals) was investigated. The research outcomes revealed that the LC_50_ of AcF and EtF extracts had gradually raised body weight for 14 days (*p* > 0.05). Similarly, there were no remarkable alternations in the complete blood count (CBC); only a slight decrease in the monocytes count (612 ± 159.80 × 10^3^ µL) in the EtF-treated group. There was a notable increase in alanine transferase (ALT) activity (36.73 ± 1.44 IU/L) in the AcF-treated group. No destructive changes were noted with the remaining biochemical parameters. Cholesterol and triglycerides non-significantly increased in the EtF group, whereas, cholesterol levels decreased significantly in the AcF group. In addition, histopathological examination reflected minor changes in AcF and EtF groups in the form of mild inflammation in the lungs and mild vacuolar degeneration in the kidneys, while no lesions were detected in the heart and liver in the same groups. Thus, the present research suggested that AcF and EtF extracts of *E. crassipes* are safe green insecticides for insect control strategies.

## 1. Introduction

*Aphis craccivora* Koch. (the bean aphid) (Hemiptera: Aphididae) is considered one of the most deleterious insects globally [1]. The bean aphid infests many parts of the bean plant (leaves, stems, twigs, pods, and flowers) [2]. In addition, these insect species cause serious economic losses in the yields (up to 100%) of a wide range of family Leguminosae [3]. Moreover, *A. craccivora* can transmit about twenty plant viruses worldwide, including the bean leaf roll virus [4]. The traditional control method for *A. craccivora* is chemical control using insecticides, but the unwise use of such chemicals resulted in secondary pests outbreaks and the resistance build-up to insecticides [5,6]. Synthetic pesticides are one of the most crucial environmental pollutants used intensively in the agricultural sector for controlling or protecting against pests and diseases. In addition, more than four million tons of pesticides are applied annually, but only 1% of these doses reach their targets [7]. Moreover, the indiscriminate usage of pesticides becomes a threat to the ecosystem. They caused direct and indirect effects on non-target organisms and have toxic residues in soils and plants, etc., which leads to environmental sustainability imbalance [8,9,10]. Botanical extracts are one of the most tested/ effective natural products against different pest species. They can act by disrupting some physiological and biological processes in the pest [11]. Botanical products service to be insect growth regulators, insecticides, repellents, and antifeedants for a large scale of insect pests due to their attribution to some active secondary metabolites, such as alkaloids, flavonoids, and terpenoids [12,13]. The extraction of such toxic bioactive phytochemicals opens the vista for further assessment as insecticidal agents, as these phytochemical compounds are considered a large reservoir of different chemical structures with a variety of biological activities [14].

*Eichhornia crassipes* (Mart) Solms (water hyacinth) (Family: Pontederiaceae) is one of the most invasive freshwater herbs globally. It has extended over many continents, especially North America, Africa, Southeast Asia, and Central America [15]. Water hyacinth grows as dense monocultures that endanger the variety of indigenous plant species in the area [16]. Furthermore, these dense mates alter the physico-chemical properties of the aquatic system. Thus, leading to alternations in the function and structure of the surrounded ecosystem through the disruption of food chains and nutrient cycle. Dense monocultures can deplete the dissolved oxygen in aquatic systems, resulting in a reduction in fish productivity [17]. Additionally, the extracts of water hyacinth reflected an insecticidal activity against many insect pests in different stages. Its acetone extract had larvicidal activity against *Achaea janata* and *Spodoptera litura* [18]. The root extract had 100% mortality when subjected to *Chironomus ramosus* larvae [19]. Moreover, acetone and ethanol fractions had a promising toxic effect on *Agrotis ipsilon* larvae [13]. Hexane and methanolic extracts had an insecticidal activity on all *Culex quinquefaciatus* stages [20].

The use of plant extracts as pesticides is widespread. The chemical composition of these plants should be detected and identified. However, in some situations, there is a lack of toxicological evaluations, especially toward non-target organisms (including mammals) [21,22]. Thus, some studies should be carried out on laboratory animals to maintain human safety. In this regard, the current research aims to identify the chemical composition of acetone and ethanol fractions extracts of *E. crassipes.* Moreover, we investigated their insecticidal effect on bean aphid adults, hematological, biochemical, and histopathological indicators of an experimental model (albino rats).

## 2. Results

### 2.1. Chemical Composition of AcF and EtF Extracts of E. crassipes

According to the GC/MS analysis data of AcF and EtF extracts, the major compounds of chemical composition of the acetone fraction extract were *n*-hexadecanoic acid (16.72%), linolenic acid (11.13%), hexadecanoic acid (9.35%), hexahydrofarnesyl acetone (9.03%), phytol (3.79%), 2-phenylundecane (2.83%), 2-phenyldodecane (2.75%), hexa-hydro-farnesol (2.60%), neophytadiene (2.39%), 2-phenyltridecane (2.24%), 12,15-octadecadiynoic acid, methyl ester (2.21%), 8-octadecenoic acid, methyl ester (2.16%), 6-phenyldodecane (2.08%), phthalic acid, and bis (2-ethylhexyl) ester (2.04%) (Table 1). The major AcF extract compounds represent about 93.72% of the total components, while the minor compounds represent about 6.28% and are listed in Table S1.

The GC/MS results of ethanol fraction extract reported thirty-one compounds that were identified. The main constituents formed about 85.12% as hexadecanoic acid, ethyl ester (7.86%), tridecane, 2-phenyl- (6.30%), 2-phenyldodecane (6.22%), hexadecanoic acid (5.51%), 6-phenyltridecane (4.99%), 3-phenyltridecane (4.47%), 5-phenyltridecane (3.66%), 4-phenyltridecane (3.56%), dodecane, 3-phenyl- (3.49%), oleic acid (3.39%), undecane, 2-phenyl- (3.37%), hexadecanoic acid, methyl ester (3.04%), dodecane, 6-phenyl- (3.01%), 9-octadecenoic acid (z)-,methyl ester (2.76%), dodecane, 4-phenyl- (2.59%), dodecane, 5-phenyl- (2.54%), diisooctyl phthalate (2.48%) (Table 1). While there were some small percentage components (14.88%), which are presented in Table S2.

### 2.2. Insecticidal Activity of AcF and EtF Extracts against Aphis craccivora

The insecticidal activity of acetone and ethanol fractionated extracts were tested against *Aphis craccivora* adults using different concentrations for each.

**Figure 1:**
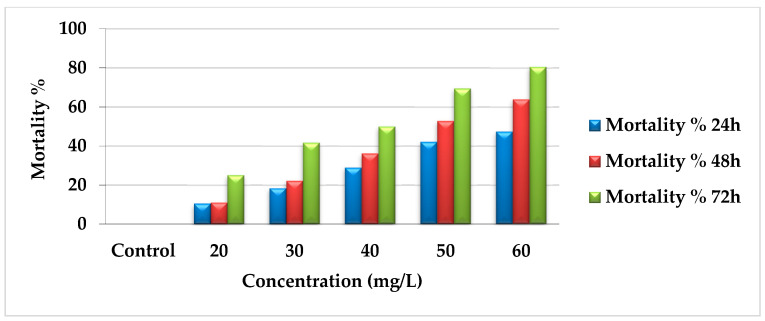
Mortality percentages of AcF extract of *E. crassipes* against *A. craccivora*.

The toxicological activity of AcF extract was tested at different concentrations (60, 50, 40, 30, and 20 mg/L). The mortality percentages increased gradually according to concentration and time gradients as follows: 47.37%, 63.9%, and 80.56% during 24, 48, and 72 h, respectively, for 60 mg/L. For 50 mg/L, the mortality was 42.11%, 52.78%, and 69.44% during 24, 48, and 72 h, respectively. At 40 mg/L, the results were 28.95%, 36.11%, and 50% for 24, 48, and 72 h, respectively. Moreover, 30 mg/L recorded 18.42%, 22.22%, and 41.67%. Finally, 20 mg/L showed 10.53%, 11.11%, and 25% for 24, 48, and 72 h, respectively (Figure 1). All the data were compared with the control group for all concentrations, which had no changes during the experiment.

**Figure 2:**
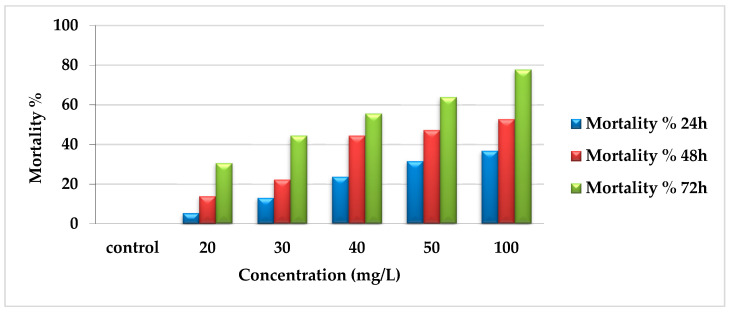
Mortality percentages of EtF extract of *E. crassipes* against *A. craccivora*.

The insecticidal effect of EtF extract reflected that the percentage of mortality was directly proportioned with concentration and time. After 24 h, mortality was 36.8%, 31.58%, 23.7%, 13.16%, and 5.3% for concentrations 100, 50, 40, 30, and 20 mg/L, respectively compared with zero percent in the control. There was a gradual increase in mortality after 48 h (52.78%, 47.22%, 44.44%, 22.22%, and 13.9%) for previously mentioned concentrations, respectively. In addition, after 72 h, the mortality percentage reached the maximum, especially with the higher concentrations (100, 50, and 40 mg/L) as 77.78%, 63.9%, and 55.56% and 44.44% and 30.56% with 30 and 20 mg/L as presented in Figure 2.

### 2.3. Efficacy of AcF and EtF Extracts against Aphis craccivora

The results of the bioassay of acetone and ethanol fraction extracts of *E. crassipes* extract against aphid adults indicated that acetone fraction extract (AcF) was more effective against *A. craccivora* during the experiment (24, 48, and 72 h) with the lowest LC_50_ values as 64, 52, and 39 mg/L, respectively. Conversely, ethanol fraction extract (EtF) had higher LC_50_ values of 140, 86, and 42 mg/L (Table 2).

### 2.4. Effect of AcF and EtF Extracts on the Number of Offspring of Aphis craccivora

**Figure 3:**
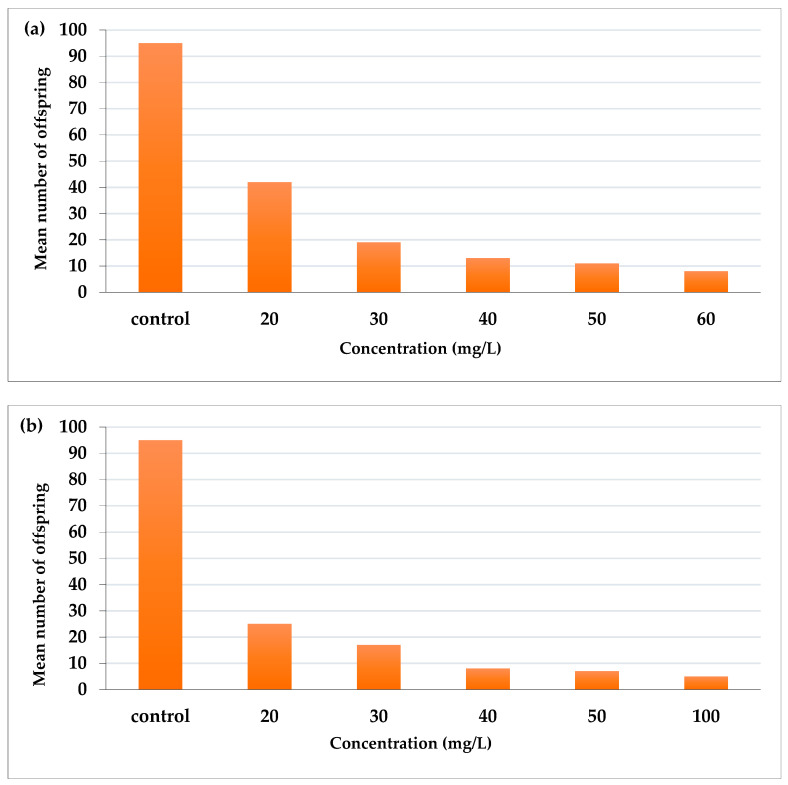
Effect of (**a**) acetone and (**b**) ethanol fractions on the fecundity of *A. craccivora* adults.

AcF extract reduced the fecundity of aphid adults more than EtF extract (Figure 3). AcF recorded the highest effect at 60 mg/L (number of offspring was 8) and 11, 13, 19, and 42 offspring for 50, 40, 30, and 20 mg/L, respectively, compared with 92 offspring in the control (Figure 3a). On the other hand, EtF extract at the highest concentration (100 mg/L) reduced the fecundity to 5 offspring then 7, 8, 17, and 25 offspring for 50, 40, 30, and 20 mg/L, respectively, compared with 95 offspring in the control (Figure 3b).

### 2.5. Effects of Exposure to LC_50_ of AcF and EtF Extracts of E. crassipes on Body Weight

**Figure 4:**
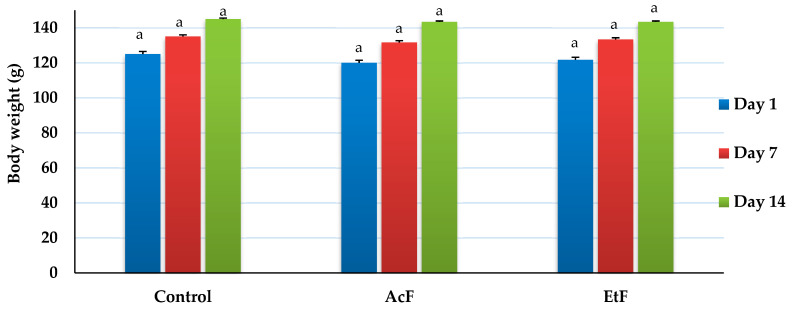
Effect of acetone and ethanol fractions of *E. crassipes* extracts in albino rat’s body weight (g) (values are means for *n* = 5 ± SE). The columns that have the same letter do not show a significant difference (*p* > 0.05) compared to the control.

Overall body weight increased with time in all groups, but there was no difference between the control and treatment groups (AcF and EtF) at any time point (14 days) (*p* > 0.05) (Figure 4). On day 1, weights were recorded as 120, 121.67 ± 1.67, and 125 g, respectively. On day 7, weights were 131.67 ± 1.67, 133.33 ± 1.67, and 135 ± 2.89 g, respectively. Whereas, on day 14, weights elevated to 143.33 ± 1.67, 143.33 ± 1.67, and 145 ± 2.89 g, respectively (*p* < 0.05).

### 2.6. Effects of Exposure to LC_50_ of AcF and EtF Extracts of E. crassipes on Hematological Indices

#### 2.6.1. Effect of LC_50_ of AcF and EtF Extracts on Differential WBC Count

After 14 days of treatment with the most potent insecticidal extracts (AcF and EtF), there was a non-significant (*p* > 0.05) effect on the values of WBC, lymphocytes, monocytes, neutrophils, eosinophils, and segmented cells with AcF-treated samples compared to the control (Table 3). Conversely, EtF-treated samples reflected a significant reduction only in the monocytes (612 ± 159.80 × 10^3^/µL) compared with the control (1006 ± 255.60 × 10^3^/µL) (*p* < 0.05) (Table 3 and Table 4).

#### 2.6.2. Effect of LC_50_ of AcF and EtF Extracts on RBC Count and Indices

Treated groups (with the LC_50_ concentration of AcF and EtF extracts) reflected non-significant (*p* > 0.05) alternations in total red blood cell count (RBC), hemoglobin, hematocrit, mean corpuscular cell volume (MCV), MCH concentration (MCHC), mean cell hemoglobin (MCH), and platelets count compared with the control group (Table 4).

### 2.7. Effects of Exposure to LC_50_ of AcF and EtF Extracts of E. crassipes on Biochemical Indices

#### 2.7.1. Effect of LC_50_ of AcF and EtF Extracts on Liver and Kidneys Functions

The results of the liver function (acid phosphatase, alkaline phosphatase, and alanine transaminase activities) and kidneys function (urea and creatinine) showed that no considerable significance was detected in the activity of acid phosphatase (ACP) with AcF and EtF extracts (3.18 ± 0.52 and 2.65 ± 0.56 U/L, respectively) when compared with the control (2.25 ± 0.29 U/L) (*p* > 0.05). Similarly, the activity of alkaline phosphatase (ALP) had no notable alternations compared with the control (342.67 ± 4.63, 329 ± 2.08, and 329.33 ± 2.40 IU/L, respectively) (*p* > 0.05). Whereas, a significant elevation (*p* < 0.05) was detected in alanine transaminase (ALT) activity in the group treated with AcF as 36.73 ± 1.44 IU/L compared with the control at 25.04 ± 1.99 IU/L (Table 5).

Urea concentration showed a non-significant effect (*p* > 0.05) in groups treated with AcF and EtF (33.4 ± 1.07 and 34.10 ± 1.31 g/dL), respectively, compared with the control (30.9 ± 0.53). Probably, creatinine had non-significant changes in AcF and EtF groups (29.05 ± 0.52 and 28.67 ± 1.91 mg/dL), respectively, compared with the control (27.37 ± 0.40 mg/dL) (*p* > 0.05) (Table 5).

#### 2.7.2. Effect of LC_50_ of AcF and EtF Extracts on Total Cholesterol and Triglycerides

**Figure 5:**
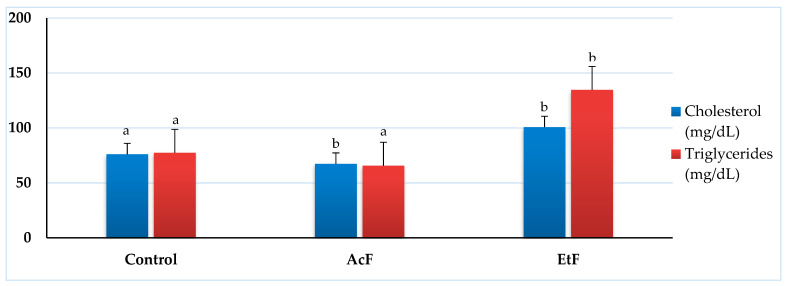
Effect of acetone and ethanol fractions of *E. crassipes* extracts on total cholesterol and triglycerides (values are means for *n* = 5 ± SE) in albino rats. The columns that have the same letter do not show a significant difference (*p* > 0.05) compared to the control.

The quantitative analysis of total cholesterol showed a significant decrease with the AcF-treated group (67.33 ± 3.84 mg/dL) (*p* < 0.05) while a significant elevation was seen with the EtF group (100.67 ± 12.67 mg/dL) when compared with the control (76 ± 3.06 mg/dL). Whereas, the triglycerides content was 77.33 ± 9.94 mg/dL in the control and had minor changes after exposure to AcF (65.67 ± 5.49 mg/dL). Similarly, the EtF treated group reflected a significant elevation in triglycerides (134.67 ± 41.91 mg/dL) (*p* < 0.05) (Figure 5).

### 2.8. Histopathological Findings and Lesion Scoring

**Figure 6:**
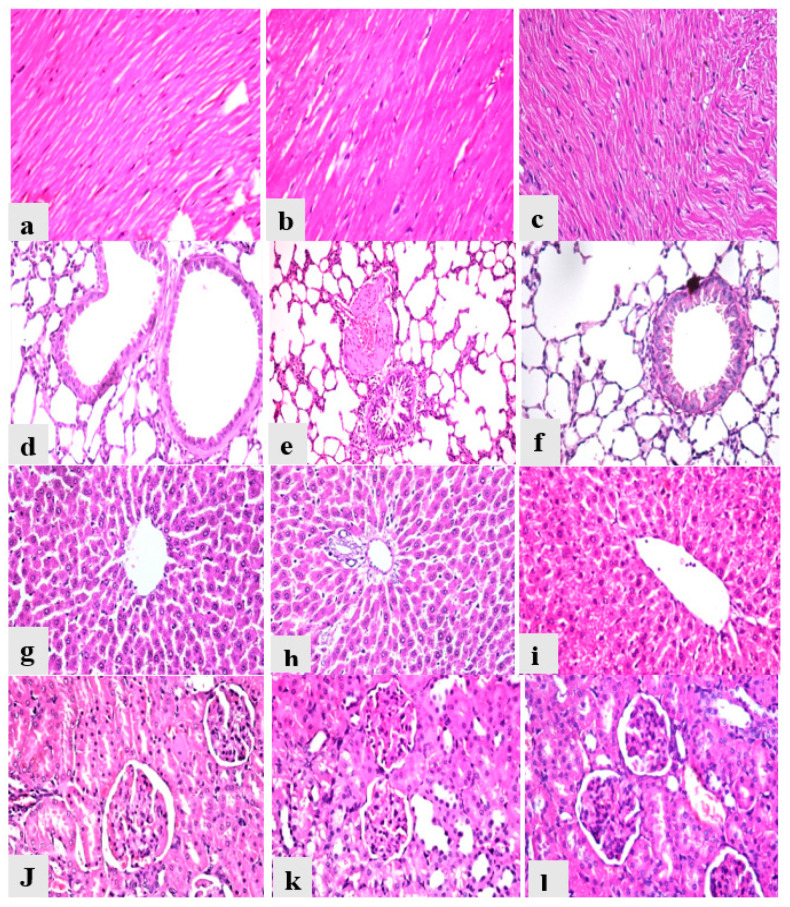
Photomicrographs of heart, lungs, liver, and kidneys from different experimental groups stained with hematoxylin and eosin. (**a**) Heart from control group (Gp I) (0). (**b**) Heart from AcF group (Gp II) (0). (**c**) Heart from EtF group (Gp III) (0). (**d**) Lung from control group (Gp I) (0). (**e**) Lung from AcF group (Gp II) (+). (**f**) Lung from EtF group (Gp III) (+). (**g**) Liver from control group (Gp I) (0). (**h**) Liver from AcF group (Gp II) (0). (**i**) Liver from EtF group (Gp III) (0). (**j**) Kidneys from the control group (Gp I) (0). (**k**) Kidneys from AcF group (+). (**l**) Kidneys from EtF group (Gp III) (+).

Heart from the control group showed normal myocardial muscles bundles with normal striations and normal nucleation with no histopathological alterations (Figure 6a); heart from the AcF and EtF groups revealed normal histology as the control group (Figure 6b,c). Lungs from the control group revealed normal bronchiole, alveoli, and interstitial tissues with no signs of inflammation (Figure 6d), while lungs from the AcF and EtF groups showed slight inflammatory signs in the form of a mildly congested blood vessel with thick muscle wall and slight peri-bronchial and interstitial tissue mononuclear cells infiltrations (Figure 6e,f). Liver from all experimental groups reported normal hepatic parenchyma with organized hepatic cords, normal blood sinusoids, and central vein (Figure 6g), and portal tract (Figure 6h,i). Kidneys from the control animals showed normal renal glomeruli, renal tubules, and interstitial tissues with no signs of degeneration (Figure 6j), while kidneys from the AcF and EtF groups showed vacuolar degeneration in some renal tubules (Figure 6k,l). Moreover, all the recorded lesions in the heart, lungs, liver, and kidneys were scored, as shown in Table 6.

## 3. Discussion

The chemical composition of water hyacinth showed the existence of some important phytochemicals, including fatty acids, alkaloids, terpenoids, glycosides, flavonoids, polyphenols, proteins, and some other metabolites [16,23,24]. The GC-mass analysis of *E. crassipes* fractionated extracts (AcF and EtF) indicated that there are many biologically active components. Among these phytochemicals large amounts of fatty acids, such as *n*-hexadecanoic acid (palmitic acid ester), linolenic acid, hexadecenoic acid (palmitic acid), myristic acid, octadecanoic acid (stearic acid), linolelaidic acid, methyl ester (polyenoic fatty acid), 8-octadecanoic acid, methyl ester (fatty acid methyl ester), 12,15-Octadecadiynoic acid, methyl ester (polyenoic fatty acid), oleic acid, ethyl linoleate (linoleic acid ethyl ester), 9-octadecanoic acid, methyl ester (polyenoic fatty acid), and lauric acid. These fatty acids can act as pesticides, anti-fungal, anti-bacterial, nematicide, insect repellent [25,26], alpha-reductase inhibitors, anti-arthritic, antioxidant, hypocholesterolemic, ant-acne [27], flavors, anti-inflammatory, anti-cancer, hepatoprotective, anti-coronary, and anti-androgenic [28,29]. Moreover, diisooctyl phthalate, 1,2-cyclohexanedicarboxylic acid diisononyl ester, phthalic acid, bis (2 ethylhexyl) ester, and phthalic acid, dinonyl ester are plasticizer compounds that can act as anti-fouling and anti-bacterial [30,31]. In addition, the results include some terpenoid compounds, such as hexahydrofarnesyl acetone (sesquiterpenoid) and hexahydrofarnesol which were found in moderate percentages in the AcF extract; they have a potential antimicrobial activity [32], insecticidal activity [33], and allopathic potentiality [34]. Phytol (diterpene) and neophytadiene both have antimicrobial activity [35], especially phytol, which has a noticeable bactericidal effect on *Staphylococcus aureus* [36,37]. Furthermore, 3,7,11,15-tetramethyl-2 hexadecen-1-ol is one of the terpenoids compounds detected in the AcF extract (in our research) and previously detected in the *E. crassipes* ethanolic extract [38]. Another group of phytochemicals detected in our research, alkaloids, including 11-hydroxy-ascididemin (pentacyclic alkaloid) (its existence in water hyacinth extracts was not mentioned in the literature) has antimicrobial potential against *Micrococcus luteus* [32]. Besides the above mentioned biologically active components, there are a wide variety of hydrocarbons in moderate percentages, especially in the EtF extract rather than the AcF extract, such as tridecane, 2-phenyl, 2-phenyldodecane, 6-phenyltridecane, 3-phenyltridecane, 5-phenyltridecane, 4-phenyltridecane, dodecane, 3-phenyl-, undecane, 2-phenyl-, dodecane, 6-phenyl, dodecane, 5-phenyl, and undecane, 3-phenyl (in EtF extract). Whereas, 2-phenyldodecane, 3-phenyldodecane, 4-phenyldodecane, 6-phenyltridecane, 3-phenyltridecane, and 5-phenyltridecane were in the AcF extract. According to our background, this is the first report for such phenylated hydrocarbons in *E. crassipes*. Similarly, there are some new compounds identified in *E. crassipes,* such as dodecyl cis-9,10-epoxyoctadecanoate, 1,2-Cyclohexanedicarboxylicacid, nonyl 4-octyl ester, 1,2-Cyclohexanedicarboxylicacid, cyclohexylmethyl nonylester, and 1,2-Cyclohexanedicarboxylicacid, nonyl 4-octyl ester. Generally, several factors influence the total amount and types of extracted phytochemicals, including the method of extraction, solvent used, the harvesting time, and the geographical origin of the plant [2].

Recently, various plant materials are being used as pesticides (insecticides). Mackled et al. [39] found that the essential oils of *Mentha piperita*, *Pinus roxburghii*, and *Rosa* spp. were highly effective against some stored product pests. Moreover, the results of Zhou et al. [40] revealed that plant derivatives of *Dracocephalum integrifolium* had a potential value against *Aphis pomi*. Extracts and fractions of *Trillium govanianum* indicate a promising insecticidal activity towards *Plutella xylostella* and *Aphis craccivora* larvae [41]. According to our data, the toxicity of AcF and EtF at different concentrations (60, 50, 40, 30, and 20 mg/L) AcF, and (100, 50, 40, 30, and 20 mg/L) for EtF for 24, 48, and 72 h, reflected that the lowest LC_50_ value (39 mg/L) was for AcF after 72 h, and 42 mg/L for EtF. These results showed that the AcF extract is more potent than EtF extract, which is in an agreement with Abdelkhalek et al. [13], who found that the acetone fraction of water hyacinth was the most effective against *Agrotis ipsilon*. Similarly, Jayanthi et al. [20] found that water hyacinth extracts have insecticidal activity against *Culex quinquefasciatus* and the mortality increased by increasing the concentrations. Conversely, Reddy et al. [42] illustrated that *parthenium hysterophorus* extract was less toxic to *A. craccivora* due to its LC_50_ value (947.87 mg/L) compared with parthenin insecticide, which had an LC_50_ value (839 mg/L). Furthermore, there was a reduction in the number of aphid progeny in samples treated with both tested extracts (AcF and EtF). These results are in an agreement with Thakshila et al. [43], who stated that the aqueous solution of *Calotropis gigantea* and *Croton laccifera* leaf extracts decreased the fecundity of *A. craccivora* significantly. Moreover, Soliman et al. [44] evaluated that the extracts of *Nerium oleander* and *Yucca glauca* (with different solvents) reduced the number of aphid’s offspring remarkably.

According to Komalamisra et al. [45], if the LC_50_ value was less than 50 mg/L, the plant extract was considered a promising insecticide, while the extracts with LC_50_ values ranged between 100–500 mg/L were to have moderate insecticidal potency. Moreover, the presence of some fatty acids in the insecticidal formulation improves its toxicological efficacy against targeted pests. Such fatty acids are environmentally safe for vertebrates, have low persistence, and do not have resistance build-up in pests [46]. For example, Yousef et al. [47] investigated the toxicological potency of linoleic acid against different larval instars of *Spodoptera littoralis* and it showed a highly significant insecticidal effect with LC_50_ values of 4.7 and 9.11 g/100 mL for 2nd and 4th larval instars, respectively. In addition, Mostafa et al. [48] found the effectiveness of three fatty acids (stearic, oleic, and linoleic acids) against the 4th larval instar of *Earias insulana*. Their results revealed that the stearic acid had the highest effect, followed by oleic and linoleic acids, and all of them had deleterious effects on the biological and biochemical parameters.

However, these criteria are dependent on the duration of exposure to the plant material and the susceptibility of the insect stage, which may change the values of LC_50_ of examined phytochemicals [49]. Thus, the presence of fatty acids (*n*-hexadecanoic acid, linolenic acid, hexadecanoic acid), phytol, and hexahydrofarnesyl acetone in AcF and EtF extracts—and according to the revealed results—makes them very promising to complete for further insecticidal formulations and field application as green insecticidal agents.

In order to confirm the eligibility of a pesticide and its introduction to the field application, the pesticide must go through a series of animal studies to determine the hazardous effects that may be expected after acute and chronic exposure [50]. Furthermore, it is mandatory to follow sets of international guidelines and maintain the national legislation [51]. The safety evaluation of the most toxic plant extracts of *E. crassipes* (acetone (AcF) and ethanol fractions (EtF) was conducted by using the lethal median concentration (LC_50_), which kills 50% of the insect population in albino rats as a mammalian model (non-target organism). There was no mortality observed in animals treated with the LC_50_ (240 and 370 mg/L for AcF and EtF, respectively), indicating the safety of the used extract. These results agree with Huma et al. [52] and Lalitha et al. [16], who found that the ethyl acetate and methanolic extracts of water hyacinth are safe with doses of 500–2000 mg/Kg body weight, which is greater than the doses used in the current research.

As body weight is considered one of the animal health indicators, our findings reflected a normal gradual increase in body weight, which indicated the safety of used extracts. These outcomes were in concurrence with that shown in some studies [53,54].

The analysis of hematological and biochemical indices indicated crucial illustrations of the changes in the physiology of the blood due to diseases or xenobiotics exposure in animal models [55]. The results showed total WBC count, lymphocyte, neutrophils, eosinophils, segmented cells, RBC count, hemoglobin, hematocrit, MCV, MCH, MCHC, and platelet counts of treated animals with AcF and EtF of water hyacinth did not reveal any remarkable alternations compared to the control group. This is in agreement with Mirza and Panchal [53], who investigated non-significant increases with most of the hematological parameters when treated with vanillic acid, and even the significant increases and reductions were in the normal range. However, our data indicated a significant decrease in monocytes numbers with the EtF extract-treated group; it cannot be considered a sign of inflammation because of their number in the reference range. Moreover, our results had a disagreement with Akintimehin et al. [56], who found that the ethanol leaf extract of *Justicia carnea* caused an increase in RBC, PVC, hemoglobin, and platelet and a significant decrease in WBC, lymphocytes, and granulocytes levels. Another controversial study indicated that a highly significant decrease in WBC, lymphocytes, and monocytes percentages occurred when animals were treated with leaves extract of *Eruca sativa* and indicated that the leaves extract had some adverse effects on the blood indices [57]. Therefore, the hematological indices indicated the safety of *E. crassipes* AcF and EtF extracts.

Among the biochemical indices evaluated in the current study were ACP, ALP, ALT, urea, creatinine, total cholesterol, and triglycerides. ACP is located in the cellular lysosome and catalyzes the peroxidation of the lysosomal membrane. Furthermore, the elevation in ACP activity in the liver may be an indicator of lysosomal membrane damage and enzymes liberation [58]. The resulting data indicated no significant changes in ACP activity. This is in disagreement with the results of Sharma et al. [58]. ALP is an enzymatic marker for the destruction of the plasma membrane and is used for testing its integrity [59]. The outcomes cleared a non-remarkable modification in the activity of ALP are consistent with Ahmad et al. [54]. Moreover, Nwosu et al. [60] had an opposite reflection as they showed that the activity of ALP was reduced in treated rats with the botanical extract. ALT is a critical marker for detecting the organ’s disruption, particularly liver and kidney. It is frequently produced in the serum when the hepatic membrane is damaged as a result of chemical exposure [61]. The presented outcomes showed a remarkable rising in the activity of ALT in animals treated with AcF compared with the control group. Nevertheless, this rising is considered to be within the normal ALT range of up to 45 IU/L as reported by Derbalah et al. [62]. This shows an agreement with other studies [54,63].

Blood urea and serum creatinine are critical indicators utilized for diagnosing kidney implementation [64]. Urea is the main nitrogen-containing protein breakdown metabolic byproduct [65]. Furthermore, creatinine is also the muscles’ metabolic byproduct. Therefore, their accumulation in blood with high levels indicates physiological implementations and kidney injury [66]. Our results revealed normal concentrations of both urea and creatinine. These findings disagree with Kanu et al. [67], who found that levels of urea and creatinine were elevated significantly throughout the exposure period to chemically synthesized pesticides reflecting its deleterious effects on the renal functions. Moreover, the data are in concordance with Thangavelu et al. [68], who showed that the *Acacia catechu* seed extract was non-toxic to kidney.

Highly accumulative levels of lipids are one of the most crucial factors in cardiovascular disorders [69]. The most serious lipids associated with these cases are total cholesterol and triglycerides [70]. In the current study, the treatment with AcF extract reflected a significant depletion in cholesterol and a non-significant reduction in triglycerides. Whereas, EtF-treated groups had a non-remarkable elevation in both cholesterol and triglycerides levels since the elevated concentrations of triglycerides are associated with a high risk of heart and blood vessels diseases. Thus, there was no cholesterol accumulation in the blood vessels of treated animals, and the levels of triglycerides were still in the normal range, so no cardiovascular dysfunction occurred. This shows an agreement with the results obtained by Arwora et al. [69] and Hadi et al. [57].

Histopathological examination of treated groups’ liver showed normal hepatic architecture without any alternations, even in the number of Kupffer cells (which activated and increased to eliminate the xenobiotics from hepatic cells). This supports the obtained biochemical results of ACP, ALP, and ALT. There was no damage observed in heart histology in AcF and EtF treated groups compared to the control group. However, the lungs had a mild inflammatory effect in groups treated with AcF and EtF extracts, the inflammation in pulmonary tissues did not indicate the toxicity of the extracts but may be a result of the existence of some active phytochemicals in the extracts. In addition, the rats did not have any respiratory discomfort during the experimental period. Kidneys tissues reflected a slight vacuolar degeneration; such few changes in the histology of AcF and EtF groups may be due to the daily intake of tested extracts [56]. Moreover, the urea and creatinine results supported normal kidney functionality. Our data were in conformity with many studies [56,60,71]. Conversely, our results disagreed with Ugagu et al. [72] and Maxwell et al. [73].

According to the aforementioned results and illustrations about the chemical composition of tested extracts, toxicological activities against *Aphis craccivora*, and the activities of hematological, biochemical parameters, and histopathological examination of different experimental animal groups, the use of AcF and EtF extracts of *E. crassipes* is considered safe as insecticidal agents in integrated pest management programs until further safety investigations are conducted.

## 4. Conclusions and Future Perspectives

Based on the obtained data, acetone and ethanol fractions of *E. crassipes* and their main elaborated components, *n*-hexadecanoic, linolenic, hexadecenoic, myristic, stearic acids, and linolelaidic acid, methyl ester and some terpenoids, alkaloids, and hydrocarbons displayed an insecticidal potency against *A. craccivora* adults and had a significant effect on aphids fecundity. The exposure of albino rats to the most potent insecticidal water hyacinth extracts (AcF and EtF) revealed normal changes in body weight, hematopoietic system, liver and kidney functions, cholesterol, and triglycerides levels. In addition, the histopathological examination showed mild effects on lungs and kidneys, which rank the extracts in the safe range for mammals. Therefore, this research study recommended that the AcF and EtF extract of water hyacinth should be considered as environmentally green insecticides and can be involved in integrated pest management programs. Furthermore, there is a need for further experiments on a field scale, some beneficial insects, and female rats’ hormones and the histopathological alterations of their reproductive system to detect more safety investigations.

## 5. Materials and Methods

### 5.1. Plant Sample and Extraction

The plants of *E. crassipes* (fresh and mature) were collected from Zhanjiang city, Guangdong province, China. The plant identification was carried out at the Botany laboratory, College of Life Science and Technology, Huazhong Agricultural University, Wuhan, China. Then, plants were thoroughly rinsed using distilled water and then dried at room temperature ~26 °C. Thereafter, an electrical blender was used for grinding the dried plants. The preparation and modifications of fractionated extracts (acetone and ethanol fractions) were prepared according to our previously published work [13]. LC_50_ concentrations of AcF and EtF (240 and 370 mg/L, respectively) extracts were used in this study. The LC_50_ concentrations used in the current research were determined according to the previous bioassay test for *Agrotis ipsilon* [13]. Since it is stronger than *Aphis craccivora*, we used their LC_50_ to be more accurate in the safety assessment tests.

### 5.2. Gas Chromatography-Mass Spectrometry (GC-MS) Analysis

The chemical composition of samples was performed using a Trace GC1310-ISQ mass spectrometer (Thermo Scientific, Austin, TX, USA) with a direct capillary column TG–5MS (30 m × 0.25 mm × 0.25 µm film thickness). The column oven temperature was initially held at 50 °C and then increased by 5 °C/min to 230 °C held for 2 min and increased to the final temperature of 290 °C by 30 °C min and held for 2 min. The injector and MS transfer line temperatures were kept at 250 and 260 °C, respectively; helium was used as a carrier gas at a constant flow rate of 1 mL/min. The solvent delay was 3 min and diluted samples of 1 µL were injected automatically using an Autosampler AS1300 coupled with GC in the split mode. EI mass spectra were collected at 70 eV ionization voltages over the range of *m*/*z* 40–1000 in full scan mode. The ion source temperature was set at 200 °C. The components were identified by comparison of their retention times and mass spectra with those of WILEY 09 and NIST 11 mass spectral databases.

### 5.3. Aphis craccivora Rearing

Parthenogenetic aphids were reared on previously planted broad beans plants (*Vicia faba* L.) on clay soil in plastic pots (7 cm diameter and 10 cm depth) under controlled laboratory conditions with a temperature of 22 ± 2 °C, relative humidity 75%, and photoperiod of 16:8 (Light: Dark) according to Soliman et al. [44] The newly ecdyced adults were selected for the experiments.

### 5.4. Efficacy/Bioassay of AcF and EtF Extracts of E. crassipes against A. craccivora

Five concentrations of each extract were prepared as 60, 50, 40, 30, and 20 mg/L for AcF and 100, 50, 40, 30, and 20 mg/L for EtF. Distilled water was used for the control groups. The insecticidal evaluation of the extracts was done using the leaf-dipping technique. Groups of ten wingless aphids were placed in Petri-dishes (9 cm diameter and 1.5 cm depth) having broad bean leaves dipped in the desired concentration for 10 s. Then, all dishes were placed in an adjusted plant growth room at 22 ± 2 °C, 75% RH, and 16:8 L:D. Five replicates were prepared for each concentration (one dish for each replicate) and each replicate had ten individuals. Accordingly, each concentration has 50 aphids. After 24, 48, and 72 h the mortality of aphid was determined by gently probing using a fine-haired brush [44]. Numbers of aphid progeny (offspring) were also assessed in order to determine the effects of tested concentrations on the fecundity of aphids. Aphids were counted by a magnifying glass.

The bioassay against *A. craccivora* was conducted at low doses and safety assessment experiments were done at higher doses (240 and 370 mg/L) based on our previous research [13].

### 5.5. Animals

This research included 15 female albino rats weighing 125–135 g. They were randomly categorized into three groups with five animals in each. Group 1: was treated with the LC_50_ AcF extract (240 mg/L). Group 2: was treated with the LC_50_ of EtF extract (370 mg/L), and Group 3 was the control group with only distilled water. Rats were housed under the optimum climatic conditions (relative humidity 25–75%, temperature 25 ± 5 °C, and photoperiod 12/12 light and dark cycles for 14 days). They were given the required doses (1 mL/mg) of body weight orally by gavage according to the LC_50_ of the insecticidal extracts of *E. crassipes.* All experimental procedures were approved and performed by the Research Ethics Committee (REC) for Animal Subject Research affiliated with Ain Shams University, Egypt (Ethics approval number: 1,8467, 2015), according to the World Health Organization (WHO) guidelines for animal care [51].

### 5.6. Body Weight and Blood Sampling

The body weight of each animal was recorded weekly during the three weeks experimental period. At the end of the experiment (14th day), rats had the last dose and starved overnight. Ultimately, they were sacrificed by cervical dislocation and the blood samples were collected through the orbital plexus into test tubes, some of them with EDTA as an anticoagulant for hematological parameters determination. The remaining tubes were without anticoagulant for the biochemical analysis.

### 5.7. Hematological and Biochemical Indices Determination

Hematological parameters of collected blood samples were analyzed using an automated hematology analyzer ABX Micros 60 (HORIBA Ltd., Montpellier, France). The blood indices were performed and included the total count of white blood cells (WBC), lymphocytes, monocytes, neutrophils, eosinophils, segmented cells, total red blood cell count (RBC), hemoglobin, hematocrit, mean cell hemoglobin (MCH), mean corpuscular cell volume (MCV), MCH concentration (MCHC), and platelets count.

Biochemical indices for liver and kidney function, such as acid phosphatase (ACP), alkaline phosphatase (ALP), alanine transaminase (ALT), urea, creatinine, total cholesterol, and triglycerides were determined using the serum of collected samples. They measured according to the protocols provided in the used commercial laboratory kits, BIODIAGNOSTIC (BIODIAGNOSTIC, Giza, Egypt) for ACP, ALP, ALT, urea, and creatinine. Cholesterol and triglycerides levels were measured with the SPINREACT kit (Ctra. Santa Coloma, 7 E-17176 SANT ESTEVE DE BAS (Barcelona, Spain).

### 5.8. Histopathological Examination

By the end of the experiment (14th day), the heart, lungs, liver, and kidneys were collected from different experimental groups and routinely processed. The paraffin-embedded blocks were sectioned at a 5-micron thickness and stained with Hematoxylin and Eosin [74] for histopathological examination by a light microscope (Olympus BX50, Tokyo, Japan).

### 5.9. Grading of Histopathological Alterations

Histopathological alterations of the heart, lungs, liver, and kidneys were graded as: (0) indicated no changes, (+), (++), and (+++) revealed mild, moderate, and severe changes, respectively [75].

### 5.10. Statistical Analysis

The mortality percentages were corrected in regard to the control using Abbott’s formula [76]. The values of lethal concentrations (LC_50_) and slope were calculated according to Finney [77], using probit analysis software. All provided data were expressed as mean ± standard error (SE) of the mean. Differences between the experimental groups were obtained by one-way ANOVA followed by Tukey’s test through the SPSS software version 22.0 (SPSS, Inc., Chicago, IL, USA) which was used for data analysis. Significant results were observed at a *p* < 0.05 level.

## Figures and Tables

**Table 1:** Chemical composition of acetone and ethanol fraction extracts of *Eichhronia crassipes*.

RT (min.)	Compound Name	RP Area (%)	MWt.	MF
Acetone Fraction Extract				
27.01	12,15-Octadecadiynoic acid, methyl ester	2.21	290	C_19_H_30_O_2_
27.64	6-phenylundecane	1.08	232	C_17_H_28_
27.73	5-phenylundecane	1.14	232	C_17_H_28_
27.97	4-phenylundecane	1.13	232	C_17_H_28_
28.48	3-phenylundecane	1.55	232	C_17_H_28_
29.37	2-phenylundecane	2.83	232	C_17_H_28_
29.85	6-Phenyldodecane	2.08	246	C_18_H_30_
29.95	5-Phenyldodecane	1.51	246	C_18_H_30_
30.23	4-Phenyldodecane	1.40	246	C_18_H_30_
30.73	3-Phenyldodecane	1.86	246	C_18_H_30_
31.23	Myristic acid	1.24	228	C_14_H_28_O_2_
31.60	2-Phenyldodecane	2.75	246	C_18_H_30_
31.96	6-phenyltridecane	1.93	260	C_19_H_32_
32.10	5-phenyltridecane	1.18	260	C_19_H_32_
32.15	Neophytadiene	2.39	278	C_20_H_38_
32.39	Hexahydrofarnesyl acetone	9.03	268	C_18_H_36_O
32.66	3,7,11,15-Tetramethyl-2 hexadecen-1-ol	1.00	296	C_20_H_40_O
32.90	3-phenyltridecane	1.82	260	C_19_H_32_
33.72	2-Phenyltridecane	2.24	260	C_19_H_32_
33.92	Hexa-hydro-farnesol	2.60	228	C_15_H_32_O
34.82	Cyclopropanenonanoic acid,2-[(2-butylcyclopropyl) methyl]-, methyl ester	1.18	322	C_21_H_38_O_2_
35.41	Hexadecanoic acid	9.35	256	C_16_H_32_O_2_
35.62	*n*-Hexadecanoic acid	16.72	256	C_16_H_32_O_2_
37.28	Linolelaidic acid, methyl ester	1.34	294	C_19_H_34_O_2_
37.40	8-Octadecenoic acid, methyl ester	2.16	296	C_19_H_36_O_2_
37.65	Phytol	3.79	296	C_20_H_40_O
38.69	Linolenic acid	11.13	278	C_18_H_30_O2
38.95	Octadecanoic acid	1.89	284	C_18_H_36_O2
44.71	Phthalic acid, bis (2 ethylhexyl) ester	2.04	390	C_24_H_38_O_4_
48.45	Phthalic acid, dinonyl ester	1.12	418	C_26_H_42_O_4_
Ethanol Fraction Extract				
26.99	Lauric acid	1.17	200	C_12_H_24_O_2_
27.62	Undecane, 6-phenyl-	1.06	232	C_17_H_28_
27.71	Undecane, 5-phenyl-	1.05	232	C_17_H_28_
27.96	Undecane, 4-phenyl-	1.26	232	C_17_H_28_
28.47	Undecane, 3-phenyl	1.59	232	C_17_H_28_
29.36	Undecane, 2-phenyl-	3.37	232	C_17_H_28_
29.85	Dodecane, 6-phenyl-	3.01	246	C_18_H_30_
29.95	Dodecane, 5-phenyl-	2.54	246	C_18_H_30_
30.23	Dodecane, 4-phenyl-	2.59	246	C_18_H_30_
30.74	Dodecane, 3-phenyl-	3.49	246	C_18_H_30_
31.25	Tetradecanoic acid	1.15	228	C_14_H_28_O_2_
31.62	2-Phenyldodecane	6.22	246	C_18_H_30_
31.99	6-Phenyltridecane	4.99	260	C_19_H_32_
32.13	5-Phenyltridecane	3.66	260	C_19_H_32_
32.40	4-Phenyltridecane	3.56	260	C_19_H_32_
32.91	3-Phenyltridecane	4.47	260	C_19_H_32_
33.76	Tridecane, 2-phenyl-	6.30	260	C_19_H_32_
34.05	Hexadecanoic acid, methyl ester	3.04	270	C_17_H_34_O_2_
35.38	Hexadecanoic acid, ethyl ester	7.86	284	C_18_H_36_O_2_
35.56	Hexadecanoic acid	5.51	256	C_16_H_32_O_2_
37.40	9-Octadecenoic acid (Z)-, methyl ester	2.76	296	C_19_H_36_O_2_
38.46	Ethyl linoleate	1.27	308	C_20_H_36_O_2_
38.57	Oleic acid	3.39	282	C_18_H_34_O_2_
44.72	Diisooctyl phthalate	2.48	390	C_24_H_38_O_4_
47.07	11-Hydroxyascididemin	1.17	299	C_18_H_9_N_3_O_2_
47.19	Dodecyl cis-9,10-epoxyoctadecanoate	1.09	466	C_30_H_58_O_3_
47.46	1,2-Cyclohexanedicarboxylicacid, nonyl 4-octyl ester	1.39	410	C_25_H_46_O_4_
47.54	1,2-Cyclohexane dicarboxylic acid diisononyl ester	1.19	424	C_26_H_48_O_4_
47.78	1,2-Cyclohexanedicarboxylicacid, cyclohexylmethyl nonylester	1.05	394	C_24_H_42_O_4_
47.90	1,2-Cyclohexanedicarboxylicacid, nonyl 4-octyl ester	1.41	410	C_25_H_46_O_4_

RT: Retention time; RP area: Relative peak area; MWt: Molecular weight; MF: Molecular formula.

**Table 2:** Efficacy of AcF and EtF extracts against *Aphis craccivora*.

Treatment	LC_50_ (mg/L)	Upper Limit	Lower Limit	Slope ± SE
AcF (24 h)	64	76	55	3.14 ± 0.28
AcF (48 h)	52	57	48	4.67 ± 0.50
AcF (72 h)	39	42	36	3.66 ± 0.21
EtF (24 h)	140	210	94	1.86 ± 0.21
EtF (48 h)	86	110	66	1.88 ± 0.10
EtF (72 h)	42	48	36	2.03 ± 0.09

LC_50_; upper limit; lower limit their confidence limits at 95%.

**Table 3:** Effect of LC_50_ concentration of acetone and ethanol fractions extracts of *E. crassipes* on differential WBC count in albino rats.

Blood Parameters	Treatment Samples		
	Control	Acetone Fraction	Ethanol Fraction
WBC (×10^3^/µL)	12.83 ± 2.02 ^a^	14 ± 2 ^a^	11.17 ± 1.9 ^a^
Lymphocytes (×10^3^/µL)	7453 ± 1348.51 ^a^	9360 ± 1440 ^a^	6555 ± 1262.71 ^a^
Monocytes (×10^3^/µL)	1006 ± 255.60 ^a^	1260 ± 180 ^a^	612 ± 159.80 ^b^
Neutrophils (×10^3^/µL)	3989 ± 369.75 ^a^	3000 ± 301.10 ^a^	3577.7 ± 536.17 ^a^
Eosinophils (×10^3^/µL)	385 ± 60.56 ^a^	380 ± 87.18 ^a^	422 ± 67.30 ^a^
Segmented (×10^3^/µL)	3860.7 ± 349.62 ^a^	2860 ± 282.13 ^a^	3466 ± 517.03 ^a^

The values are means ± SE (*n* = 5). Means that have the same letter do not show a significant difference (*p* > 0.05) compared to the control.

**Table 4:** Effect of LC_50_ concentration of acetone and ethanol fractions extracts of *E. crassipes* on differential RBC count in albino rats.

Blood Parameters	Treatment Samples		
	Control	Acetone Fraction	Ethanol Fraction
RBC (×10^6^/µL)	5.80 ± 0.47 ^a^	5.57 ± 0.35 ^a^	5.40 ± 0.35 ^a^
Hemoglobin (g/Dl)	13.87 ± 0.49 ^a^	14.13 ± 0.52 ^a^	13.43 ± 0.24 ^a^
Hematocrit (%)	41 ± 1 ^a^	41.90 ± 1.63 ^a^	40 ± 1 ^a^
MCV (fL)	71.60 ± 5.80 ^a^	75.60 ± 3.19 ^a^	74.80 ± 5.86 ^a^
MCH (ρg)	24.29 ± 2.42 ^a^	25.52 ± 1.34 ^a^	25.1 ± 1.76 ^a^
MCHC (g/dL)	33.83 ± 0.69 ^a^	33.73 ± 0.37 ^a^	33.60 ± 0.29 ^a^
Platelets (×10^3^/µL)	246.67 ± 4.81 ^a^	298 ± 4.58 ^a^	296.67 ± 6.36 ^a^

The values are means ± SE (*n*= 5). Means that have the same letter do not show a significant difference (*p* > 0.05) compared to the control.

**Table 5:** Effect of LC_50_ concentration of acetone and ethanol fractions extracts of *E. crassipes* on liver and kidney functions in albino rats.

**Treatment Samples**	**ACP** (**U/L**)	**ALP** (IU/L)	**ALT** (IU/L)	**Urea** (g/dL)	**Creatinine** (mg/dL)
Control	2.25 ± 0.29 ^a^	329.33 ± 2.40 ^a^	25.04 ± 1.99 ^a^	30.9 ± 0.53 ^a^	27.37 ± 0.40 ^a^
Acetone fraction	3.18 ± 0.52 ^a^	342.67 ± 4.63 ^a^	36.73 ± 1.44 ^b^	33.4 ± 1.07 ^a^	29.05 ± 0.52 ^a^
Ethanol fraction	2.65 ± 0.56 ^a^	329 ± 2.08 ^a^	35.13 ± 4.19 ^a^	34.10 ± 1.31 ^a^	28.67 ± 1.91 ^a^

The values are means ± SE (*n* = 5). Means that have the same letter do not show a significant difference (*p* > 0.05) compared to the control.

**Table 6:** Grading of histopathological lesions in heart, lungs, liver, and kidneys in all experimental groups.

Organs		Grades of the Lesions			
		0 (Negative)	+ (Mild)	++ (Moderate)	+++ (Sever)
**Control group**	**Heart**	**√**			
	**Lungs**	**√**			
	**Liver**	**√**			
	**Kidneys**	**√**			
**AcF group**	**Heart**	**√**			
	**Lungs**		**√**		
	**Liver**	**√**			
	**Kidneys**		**√**		
**EtF group**	**Heart**	**√**			
	**Lungs**		**√**		
	**Liver**	**√**			
	**Kidneys**		**√**		

## Data Availability

Not applicable.

## Supplementary Materials

The following supporting information can be downloaded at: https://www.mdpi.com/article/10.3390/toxins14050327/s1, Table S1: Minor phytochemicals of acetone fraction extract of *Eichhornia crassipes*; Table S2: Minor phytochemicals of ethanol fraction extract of *Eichhornia crassipes.*
