# Supplementary material for: The Toxicity of Eichhornia crassipes Fractionated Extracts against Aphis craccivora and Its Safety in Albino Rats
Source: Toxins (Basel). 2022 May 4;14(5):327. doi: 10.3390/toxins14050327 (PMC9144080; doi:10.3390/toxins14050327)
Supplement: Supplementary file 1 [file toxins-14-00327-s001.zip › toxins-1657287-supplementary.pdf]

**Table S1.** Minor phytochemicals of acetone fraction extract of *Eichhornia crassipes.*

| RT (min.) | Compound Name | RP Area (%) | MWt. | MF |
|---|---|---|---|---|
| 23.51 | Dodecane | 0.51 | 170 | $C_{12}H_{26}$ |
| 31.37 | 1,3,5-Triazine-2,4-diamine,6-chloro-N-ethyl- | 0.56 | 173 | $C_5H_8ClN_5$ |
| 33.04 | 3,7,11,15-Tetramethyl-2-hexadecen-1-ol | 0.70 | 296 | $C_{20}H_{40}O$ |
| 33.24 | Pentadecanoic acid | 0.76 | 242 | $C_{15}H_{30}O_2$ |
| 34.03 | Pentadecanoic acid, 14-methyl-, methyl ester | 0.98 | 270 | $C_{17}H_{34}O_2$ |
| 37.86 | Octadecanoic acid, methyl ester | 0.55 | 298 | $C_{19}H_{38}O_2$ |
| 42.39 | Hexanedioic acid, dioctyl ester | 0.74 | 370 | $C_{22}H_{42}O_4$ |
| 48.86 | Phthalic acid, dinonyl ester | 0.86 | 418 | $C_{26}H_{42}O_4$ |
| 49.28 | 1,2-Benzenedicarboxylic acid,dinonyl ester | 0.62 | 418 | $C_{26}H_{42}O_4$ |

RT: Retention time; RP area: Relative peak area; MWt: Molecular weight; MF: Molecular formula.

**Table S2.** Minor phytochemicals of ethanol fraction extract of *Eichhornia crassipes.*

| RT (min.) | Compound Name | RP Area (%) | MWt. | MF |
|---|---|---|---|---|
| 15.75 | DL-Arabinose | 0.19 | 150 | $C_5H_{10}O_5$ |
| 15.80 | 1,2,3-Propanetriol | 0.08 | 92 | $C_3H_8O_3$ |
| 15.86 | d-Mannose | 0.18 | 180 | $C_6H_{12}O_6$ |
| 17.38 | 1,3,3-Trideuterio-endo-6-hydroxy-9-oxabicyclo (3.3.1) nonan 2-one | 0.23 | 156 | $C_8H_9D_3O_3$ |
| 17.45 | trans-2-undecenoic acid | 0.35 | 184 | $C_{11}H_{20}O_2$ |
| 18.02 | 2(3H)-Furanone, 3-butyldihydro- | 0.66 | 142 | $C_8H_{14}O_2$ |
| 21.35 | Stearic acid,3 (octadecyloxy) propyl ester | 0.09 | 594 | $C_{39}H_{78}O_3$ |
| 24.18 | 1-Tetradecanol | 0.09 | 214 | $C_{14}H_{30}O$ |
| 25.25 | 2-tert-Butyl-4 isopropyl-5-meth ylphenol | 0.58 | 206 | $C_{14}H_{22}O$ |
| 25.37 | Acrylothiamide, 2-(4-chlorophenyl)-3 (methoxy carbonylmethylamino)-N-benzyl- | 0.27 | 374 | $C_{19}H_{19}ClN_2O_2S$ |
| 28.96 | 1H Cyclopropa[3,4] benz[1,2-e] azulene 4a,5,7b,9,9a(1aH)-pentol, | 0.08 | 534 | $C_{28}H_{38}O_{10}$ |
| 29.07 | Benzene, 1,4-didecyl- | 0.09 | 358 | $C_{26}H_{46}$ |

| | | | | |
|---|---|---|---|---|
| 29.20 | 11-Benzylheneicosane | 0.14 | 386 | $C_{28}H_{50}$ |
| 32.64 | Tridecanoic acid, 13-formyl-, ethyl ester | 0.29 | 270 | $C_{16}H_{30}O_3$ |
| 33.03 | Isopropyl linoleate | 0.14 | 322 | $C_{21}H_{38}O_2$ |
| 33.26 | Pentadecanoic acid | 0.20 | 242 | $C_{15}H_{30}O_2$ |
| 33.39 | Ethyl 14-oxotetradecanoate | 0.32 | 270 | $C_{16}H_{30}O_3$ |
| 34.82 | 1,2-Benzenedicarboxylic acid, butyl octyl ester | 0.40 | 334 | $C_{20}H_{30}O_4$ |
| 34.93 | Ethyl 9-hexadecenoate | 0.38 | 282 | $C_{18}H_{34}O_2$ |
| 35.13 | 9-Hexadecenoic acid, eicosyl ester, (Z)- | 0.19 | 534 | $C_{36}H_{70}O_2$ |
| 36.95 | 6,9,12-Octadecatrienoic acid, methyl ester | 0.12 | 292 | $C_{19}H_{32}O_2$ |
| 37.26 | Methyl linoleate | 0.97 | 294 | $C_{19}H_{34}O_2$ |
| 37.49 | Methyl trans-8 octadecenoate | 0.34 | 296 | $C_{19}H_{36}O_2$ |
| 37.85 | Methyl stearate | 0.76 | 298 | $C_{19}H_{38}O_2$ |

| | | | | |
|---|---|---|---|---|
| 38.92 | Octadecanoic acid, 2,3-dihydroxypropyl ester | 0.71 | 358 | $C_{21}H_{42}O_4$ |
| 39.04 | Octadecanoic acid, ethyl ester | 0.59 | 312 | $C_{20}H_{40}O_2$ |
| 42.42 | Lucenin 2 | 0.10 | 610 | $C_{27}H_{30}O_{16}$ |
| 46.35 | 4-O-Methylphorbol 12,13-didecanoate | 0.31 | 686 | $C_{41}H_{66}O_8$ |
| 46.49 | 3-Hydroxyspirost-8-en-11-one | 0.10 | 428 | $C_{27}H_{40}O_4$ |
| 46.60 | Propane, 1,3-bis(octadecyloxy)- | 0.18 | 580 | $C_{39}H_{80}O_2$ |
| 46.71 | Dodecyl 8-(3-octyl-2-oxiranyl) octanoate | 0.72 | 299 | $C_{16}H_{13}NO_5$ |
| 46.83 | Dodecyl cis-9,10-epoxyoctadecanoate | 0.62 | 466 | $C_{30}H_{58}O_3$ |
| 46.98 | 1,3-dioctadecyloxy-propane | 0.16 | 580 | $C_{39}H_{80}O_2$ |
| 47.31 | Dodecyl 8-(3-octyl-2-oxiranyl) octanoate | 0.28 | 466 | $C_{30}H_{58}O_3$ |
| 47.65 | 1,2-Cyclohexanedicarboxylic acid, dinonyl ester | 0.35 | 424 | $C_{26}H_{48}O_4$ |
| 48.01 | Propane, 1,3-bis (octadecyloxy)- | 0.40 | 580 | $C_{39}H_{80}O_2$ |

| | | | | |
|---|---|---|---|---|
| 48.14 | 1,2-Cyclohexanedicarboxylic acid, 2-ethylhexyl nonyl ester | 0.98 | 410 | $C_{25}H_{46}O_4$ |
| 48.27 | 1,2-Cyclohexanedicarboxylic acid, dinonyl ester | 0.78 | 424 | $C_{26}H_{48}O_4$ |
| 48.55 | Octadecane, 1,1'- [1,3 propanediylbis(oxy)] bis- (CAS) | 0.78 | 580 | $C_{39}H_{80}O_2$ |
| 48.70 | Octadecane, 1,1'-[1,3-propanediylbis(oxy)] bis- | 0.33 | 580 | $C_{39}H_{80}O_2$ |
| 48.96 | 2-[Hydroxy(4'-nitrophenyl) methyl]-11-phenoxyprop-2-en-1-one | 0.35 | 299 | $C_{16}H_{13}NO_5$ |

RT: Retention time; RP area: Relative peak area; MWt: Molecular weight; MF: Molecular formula.
